# Supplementary figures and images for: Lhx2 is a progenitor-intrinsic modulator of Sonic Hedgehog signaling during early retinal neurogenesis
Source: eLife. 2022 Dec 2;11:e78342. doi: 10.7554/eLife.78342 (PMC9718532; doi:10.7554/eLife.78342)

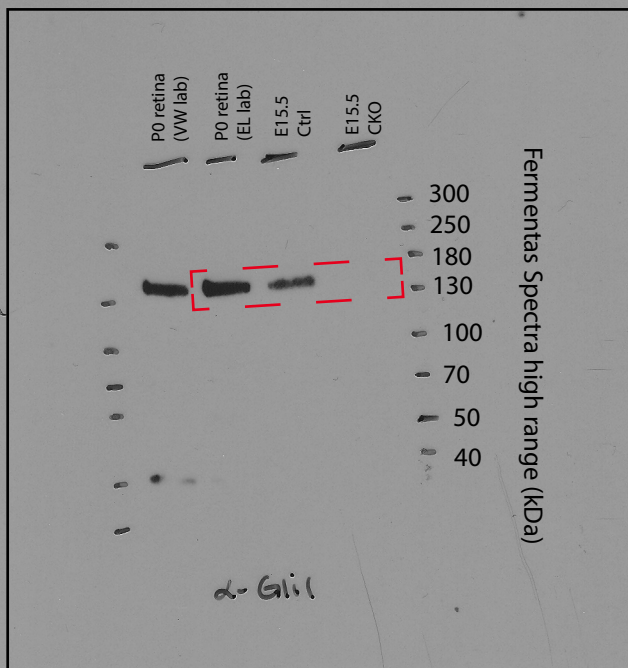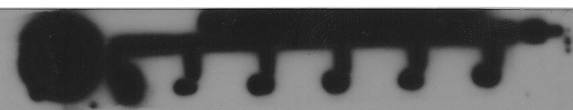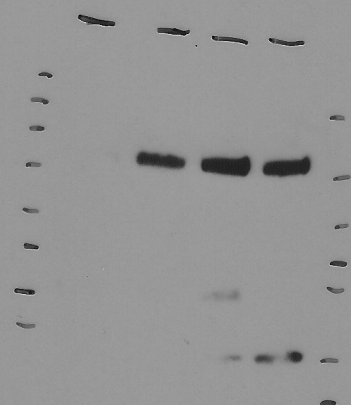

$\alpha$ -Gli1

$\alpha$ -Gli1

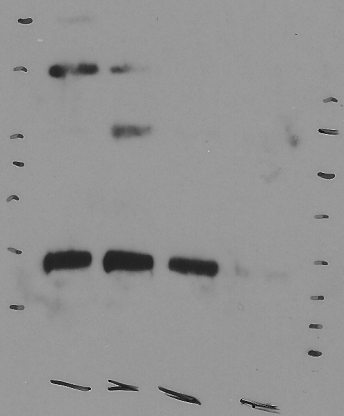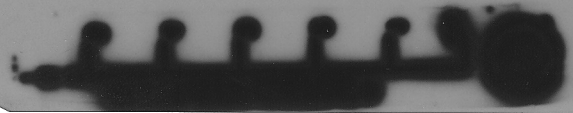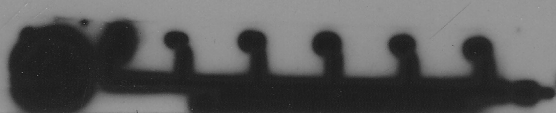

Supplement: Figure 1—source data 1. — Filenames containing the word ‘unedited’ show multiple exposures of the same blot and antibody. Filenames containing the word ‘labeled’ show the exposure used in Figure 1 as indicated by the black boxes. The red boxes indicate the portions of the blots shown in Figure 1. The sizes of the protein standards are shown in kiloDaltons (kDa). [file elife-78342-fig1-data1.zip › Figure 1-source data 1/Gli1 antibody_labeled.pdf]

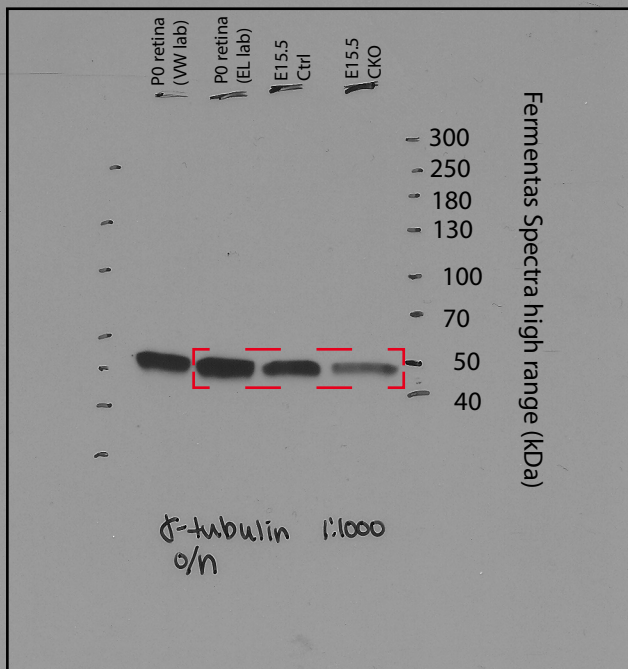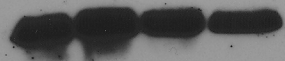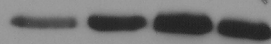

Supplement: Figure 1—source data 1. — Filenames containing the word ‘unedited’ show multiple exposures of the same blot and antibody. Filenames containing the word ‘labeled’ show the exposure used in Figure 1 as indicated by the black boxes. The red boxes indicate the portions of the blots shown in Figure 1. The sizes of the protein standards are shown in kiloDaltons (kDa). [file elife-78342-fig1-data1.zip › Figure 1-source data 1/Gamma tubulin antibody_labeled.pdf]

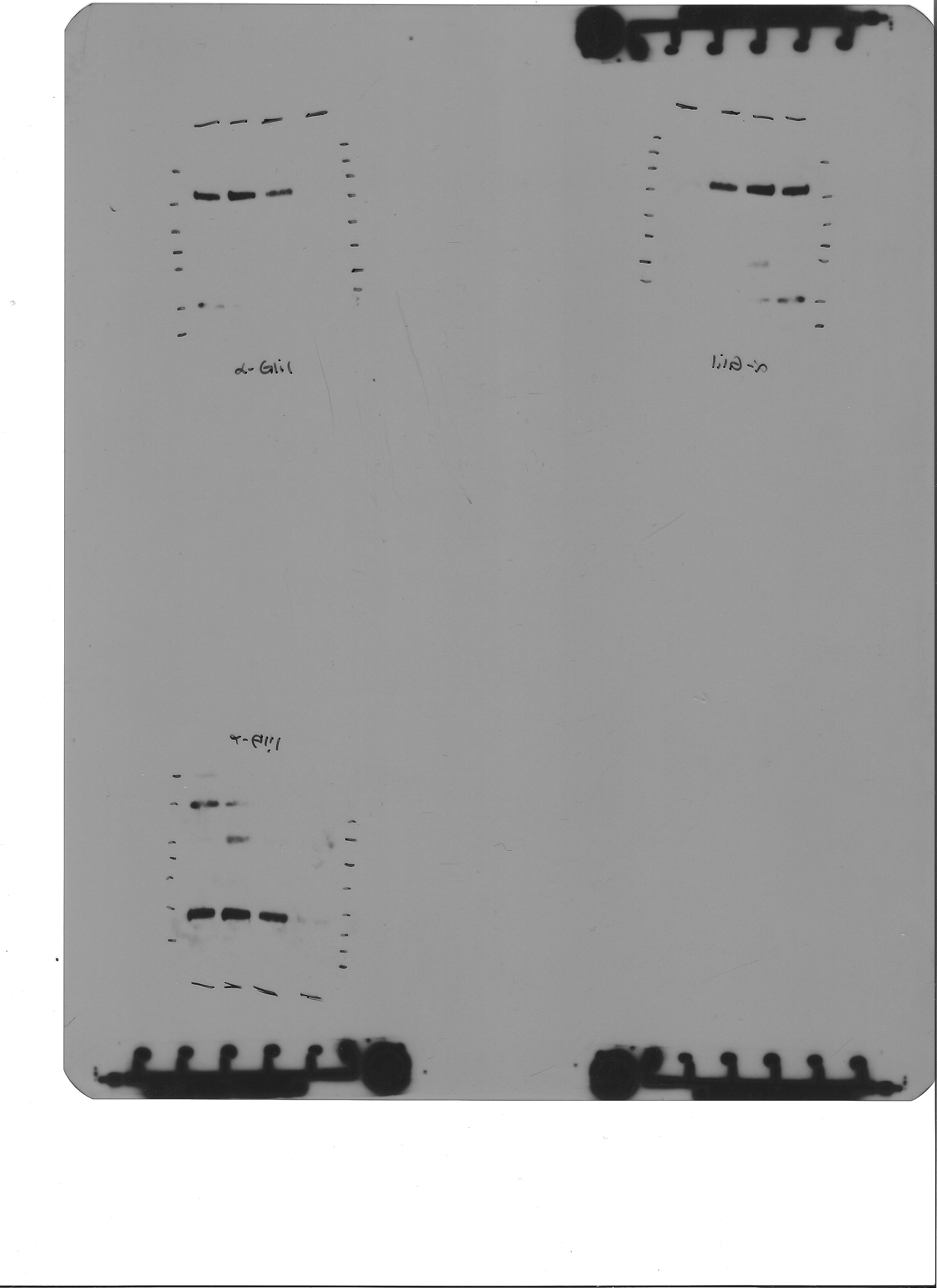

Supplement: Figure 1—source data 1. — Filenames containing the word ‘unedited’ show multiple exposures of the same blot and antibody. Filenames containing the word ‘labeled’ show the exposure used in Figure 1 as indicated by the black boxes. The red boxes indicate the portions of the blots shown in Figure 1. The sizes of the protein standards are shown in kiloDaltons (kDa). [file elife-78342-fig1-data1.zip › Figure 1-source data 1/Gli1 antibody_unedited.tiff]

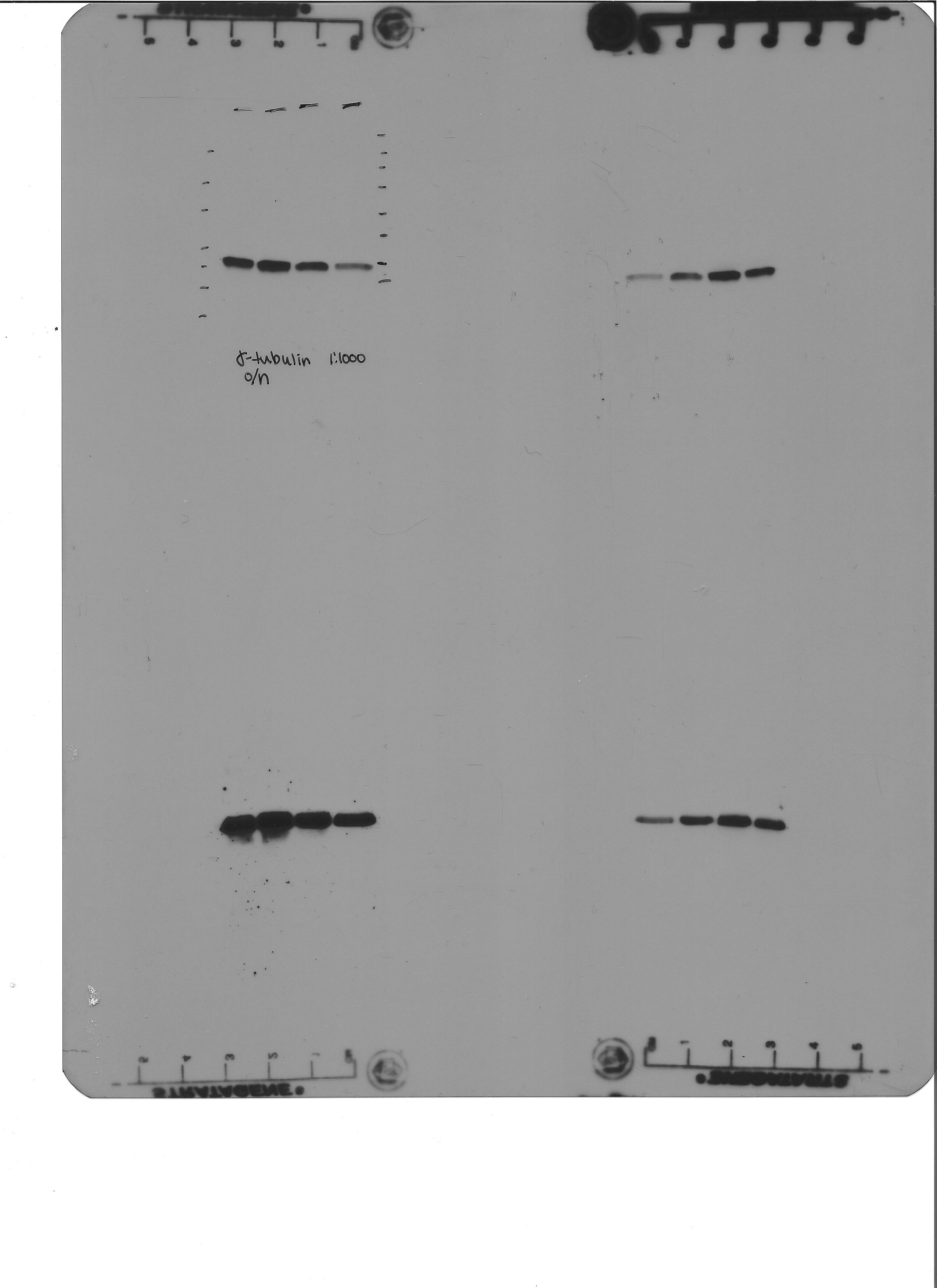

Supplement: Figure 1—source data 1. — Filenames containing the word ‘unedited’ show multiple exposures of the same blot and antibody. Filenames containing the word ‘labeled’ show the exposure used in Figure 1 as indicated by the black boxes. The red boxes indicate the portions of the blots shown in Figure 1. The sizes of the protein standards are shown in kiloDaltons (kDa). [file elife-78342-fig1-data1.zip › Figure 1-source data 1/Gamma tubulin antibody_unedited.tiff]

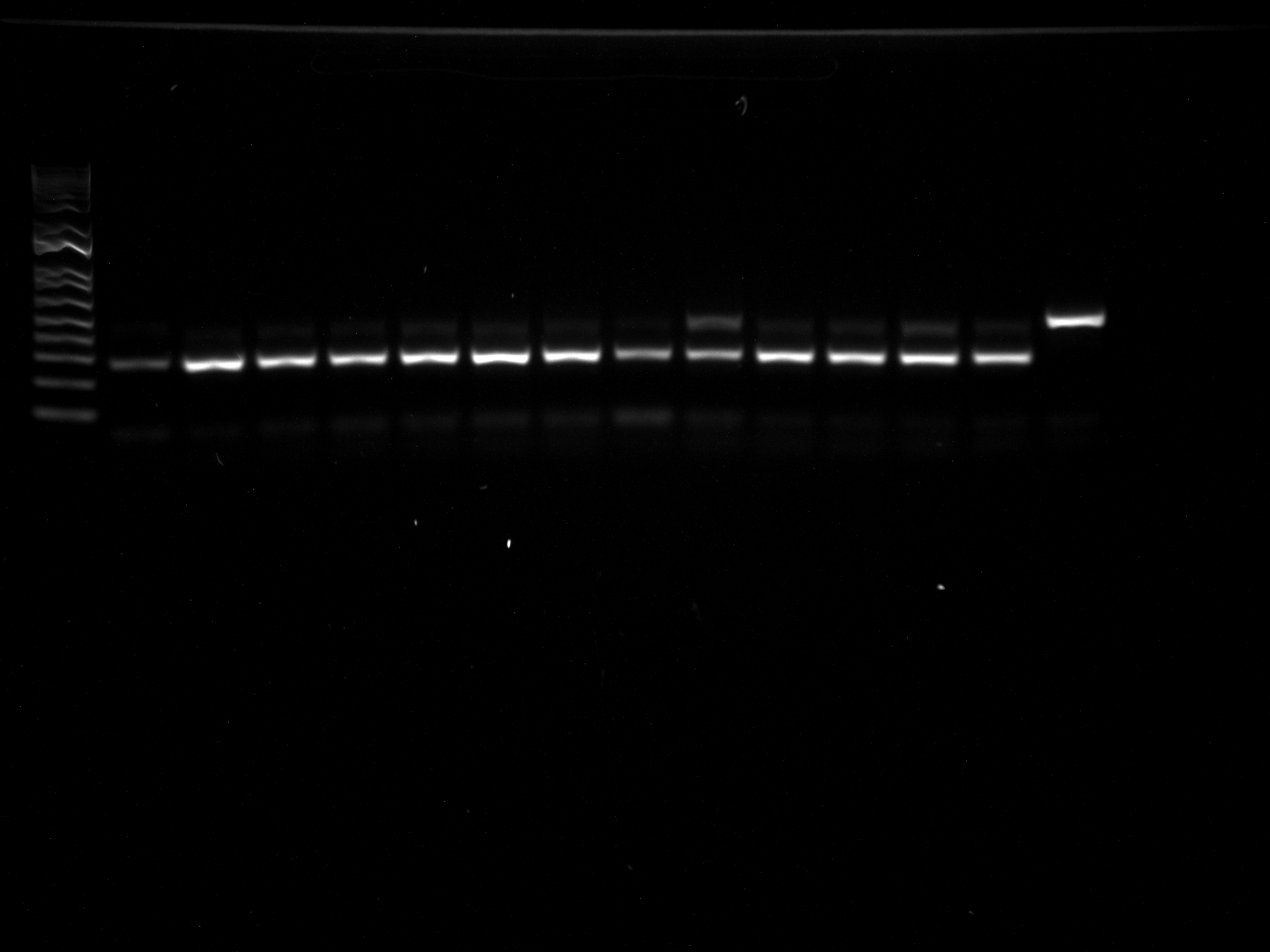

Supplement: Figure 6—figure supplement 1—source data 1. — File with the word ‘unedited’ shows the original gel capture. File with the word ‘labeled’ shows the fragment sizes of the DNA standards in base pairs (bp). The red box indicates the portion of the gel shown in Figure 6—figure supplement 1. The sizes of the non-recombined and deleted Ptch1 mRNAs are shown in base pairs (bp). [file elife-78342-fig6-figsupp1-data1.zip › Figure 6-figure supplement 1-source data 1/Ptch1 RTPCR source data_unedited.tif]

Invitrogen 1 Kb plus  
DNA ladder

**Ptch1 CKO**

**dCKO**

**Ctrl**

length (bp)  
1000  
850  
650  
500  
400  
300  
200  
100

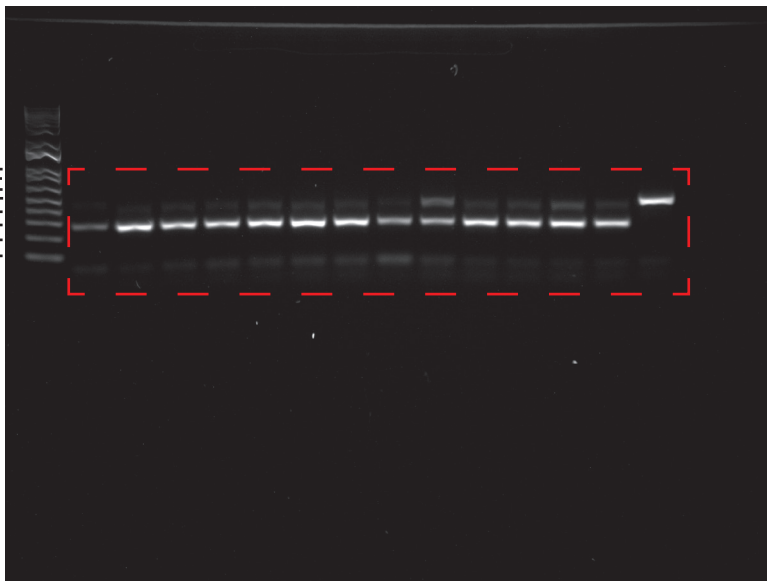

— flox (463 bp)  
— Δ (273 bp)

Supplement: Figure 6—figure supplement 1—source data 1. — File with the word ‘unedited’ shows the original gel capture. File with the word ‘labeled’ shows the fragment sizes of the DNA standards in base pairs (bp). The red box indicates the portion of the gel shown in Figure 6—figure supplement 1. The sizes of the non-recombined and deleted Ptch1 mRNAs are shown in base pairs (bp). [file elife-78342-fig6-figsupp1-data1.zip › Figure 6-figure supplement 1-source data 1/Ptch1 RTPCR_labeled.pdf]
